# The HIV-derived protein Vpr_52-96_ has anti-glioma activity *in vitro* and *in vivo*

**DOI:** 10.18632/oncotarget.9787

**Published:** 2016-06-02

**Authors:** Jens Kübler, Stefanie Kirschner, Linda Hartmann, Grit Welzel, Maren Engelhardt, Carsten Herskind, Marlon R. Veldwijk, Christian Schultz, Manuela Felix, Gerhard Glatting, Patrick Maier, Frederik Wenz, Marc A. Brockmann, Frank A. Giordano

**Affiliations:** ^1^ Department of Radiation Oncology, Universitätsmedizin Mannheim, Medical Faculty Mannheim, Heidelberg University, Mannheim, Germany; ^2^ Department of Neuroradiology, Universitätsmedizin Mannheim, Medical Faculty Mannheim, Heidelberg University, Mannheim, Germany; ^3^ Centre for Biomedicine and Medical Technology Mannheim (CBTM), Institute of Neuroanatomy, Medical Faculty Mannheim, Heidelberg University, Mannheim, Germany; ^4^ Medical Radiation Physics/Radiation Protection, Universitätsmedizin Mannheim, Medical Faculty Mannheim, Heidelberg University, Mannheim, Germany; ^5^ Department of Neuroradiology, University Medical Center of the Johannes Gutenberg University Mainz, Mainz, Germany

**Keywords:** glioblastoma, viral protein R, irradiation, MGMT, temozolomide

## Abstract

Patients with actively replicating human immunodeficiency virus (HIV) exhibit adverse reactions even to low irradiation doses. High levels of the virus-encoded viral protein R (Vpr) are believed to be one of the major underlying causes for increased radiosensitivity. As Vpr efficiently crosses the blood-brain barrier and accumulates in astrocytes, we examined its efficacy as a drug for treatment of glioblastoma multiforme (GBM).

*In vitro*, four glioblastoma-derived cell lines with and without methylguanine-DNA methyltransferase (MGMT) overexpression (U251, U87, U251-MGMT, U87-MGMT) were exposed to Vpr, temozolomide (TMZ), conventional photon irradiation (2 to 6 Gy) or to combinations thereof. Vpr showed high rates of acute toxicities with median effective doses of 4.0±1.1 μM and 15.7±7.5 μM for U251 and U87 cells, respectively. Caspase assays revealed Vpr-induced apoptosis in U251, but not in U87 cells. Vpr also efficiently inhibited clonogenic survival in both U251 and U87 cells and acted additively with irradiation. In contrast to TMZ, Vpr acted independently of MGMT expression.

Dose escalation in mice (n=12) was feasible and resulted in no evident renal or liver toxicity. Both, irradiation with 3×5 Gy (n=8) and treatment with Vpr (n=5) delayed intracerebral tumor growth and prolonged overall survival compared to untreated animals (n=5; p*_3×5 Gy_*<0.001 and p*_Vpr_*=0.04; log-rank test).

Our data show that the HIV-encoded peptide Vpr exhibits all properties of an effective chemotherapeutic drug and may be a useful agent in the treatment of GBM.

## INTRODUCTION

Patients infected with the human immunodeficiency virus (HIV) are highly predisposed to develop AIDS-defining and non-AIDS-defining malignancies [1]. Although many of these tumors (such as Kaposi's sarcoma or non-Hodgkin lymphoma) are highly radiosensitive, radiotherapy (RT) of these tumors may be challenging in HIV-infected patients as they exhibit increased rates of adverse effects even with highly active antiretroviral therapy [2–6].

The agent causing this increase in radiosensitivity is believed to be a virus-encoded peptide termed Viral protein R (Vpr) [7]. Vpr can be found virion-associated, intracellular and extracellular in cerebrospinal fluid (CSF), as well as in plasma of HIV-infected patients [8]. Over the last decade, various activities have been ascribed to the peptide: First, Vpr has been reported to cause efficient cell cycle arrest in G2- and M- phases, where cells are most radiosensitive [9–13]. Second, Vpr induces activation of caspase 3, 7, 8 and 9 [8, 14], which most likely is mediated via permeabilization of the mitochondrial membrane, uncoupling of the respiration chain and release of pro-apoptotic proteins. Third, Vpr has been found to bind to host cellular DNA, hereby inducing double strand breaks and genomic instability [15, 16], which putatively resembles a conserved mechanism to allow integrase-independent integration of HIV [17].

In addition, Vpr creates cellular damage profiles similar to cisplatin [18] and alkylating agents [15] and the use of the protein as a cancer treatment had been proposed many years ago and the mechanisms behind its effects have been explored since [19]. Various tumor entities are sensitive to Vpr, including neuroblastoma (LAN-2), lymphoma (U937), WHO grade III astrocytoma (U373), cervical cancer (HeLa), liver (HepG2), kidney (293T), melanoma (B16.F10) and leukemia (Jurkat T) cells [8, 20–22]. Consequently, first successful approaches to explore the therapeutic efficacy of Vpr were made in gene transfer studies, where Vpr over-expression inhibited growth of melanoma (B78/H1) and oral squamous cell carcinoma cell lines (AT-84) *in vitro* and *in vivo* [10, 23, 24]. The protein was also already employed as a local therapeutic agent by Siddiqui and colleagues, who injected Vpr into mammary carcinoma allografts in mice and observed efficient tumor regression with development of central necrosis in Vpr-treated tumors [18].

Due to the potential radiosensitizing ability in HIV-infected humans and its alkylator-like cellular damage signature, we hypothesized Vpr to have potential as a novel agent for the treatment of high-grade gliomas, and specifically glioblastoma multiforme (GBM). Whereas both RT and alkylating agents (i.e. temozolomide, TMZ) are considered as the current standard for adjuvant treatment of GBM [25], only modestly improved outcomes are achievable with TMZ in this tumor entity with reported median survival rates ranging between 12 and 14 months [26, 27]. However, in patients (over)expressing the O^6^-Methylguanine-DNA-Methyltransferase (MGMT) gene due to promoter de-methylation, prognosis is even more devastating as to date there is no systemic therapy option with proven efficacy [25, 28].

In the present work we investigated the effects of Vpr on U251, U251-MGMT, U87 and U87-MGMT cells alone or in combination with irradiation and TMZ *in vitro*. Furthermore, we assessed whether the peptide is tolerated by mice using a 3+3 dose escalation scheme. Finally, we tested the *in vivo* efficacy of Vpr using a clinically relevant orthotopic xenograft mouse model of malignant glioma.

## RESULTS

### Vpr shows high acute toxicity *in vitro*

**Figure 1:**
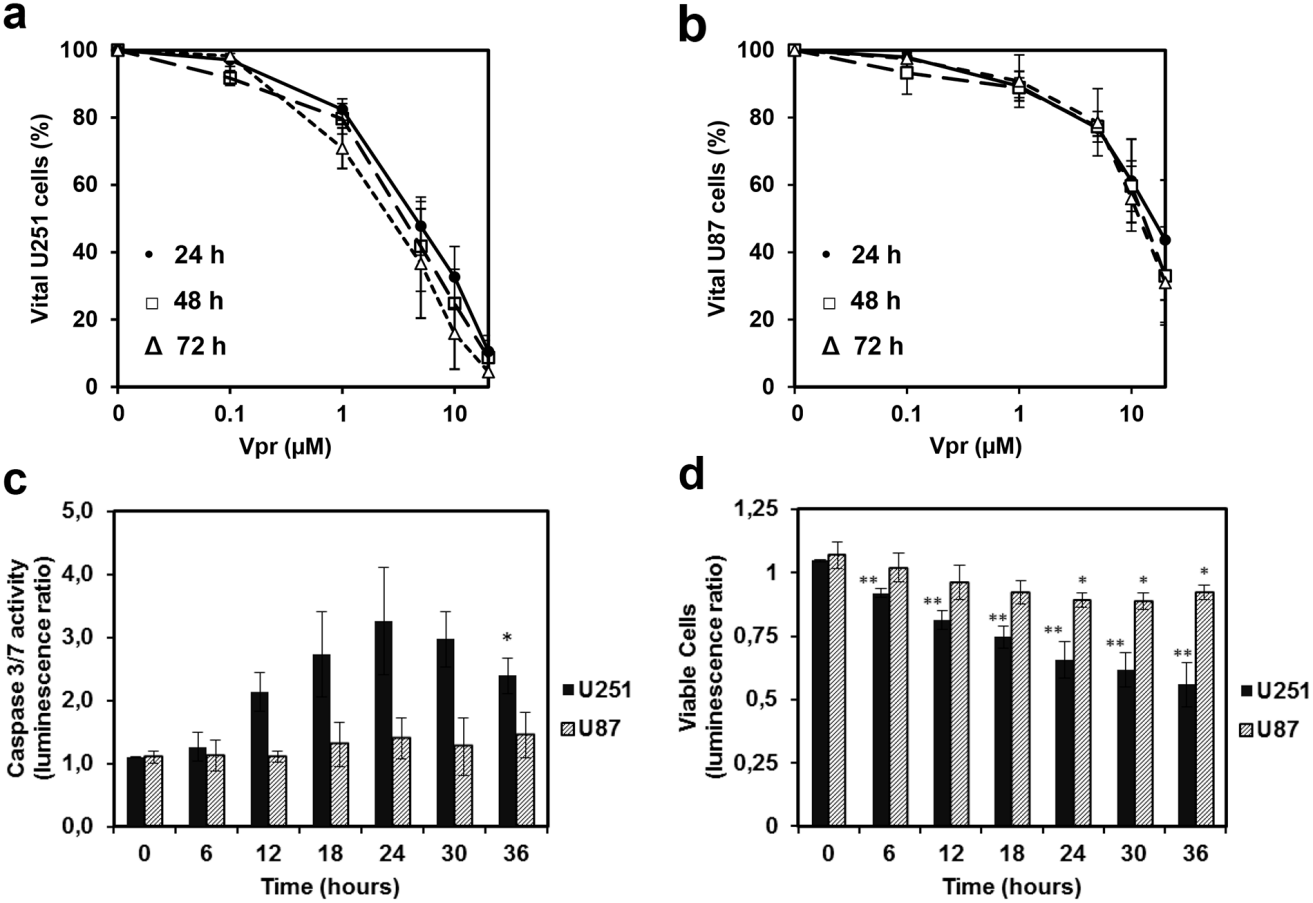
Vpr exhibits cytotoxic effects in a dose dependent manner and induces apoptosis in glioma cell lines Cell viability after treatment of U251 **a.** and U87 cells **b.** with Vpr (0/0.1/1/5/10/20 μM) for 24 (•), 48 (□) and 72(Δ) h. **c.** Effector caspase activity after 12 h. **d.** Assessment of vital cells by the luminescence assay, which was performed in parallel to the caspase activation assay. The ratio was calculated from treated (5 μM) and untreated cells and represents the amount of metabolically active cells. Results are presented as mean±SD.

U251 and U87 cells were treated with varying concentrations of medium-dissolved Vpr (0.1 to 20 μM) for 24 to 72 h (Figure 1a and 1b). Here, Vpr exhibited a concentration-dependent toxicity both in U251 and U87 cells in MTT assays. Although both cell lines were equally susceptible to low doses of Vpr (portion of vital cells at 1 μM Vpr: 82±3% for U251 cells and 89±4 % for U87 cells), U251 showed increased sensitivity towards higher doses (fraction of vital cells at 20 μM Vpr: 11±5 % for U251 cells and 44±18 % for U87 cells) compared to untreated controls. The median effective doses (EC_50_) were 4.0±1.1 μM for U251 and 15.7±7.5 μM for U87 cells (after 24 hours). When comparing different treatment and incubation periods, there were no detectable differences after 24, 48 and 72 h of Vpr exposition, indicating a predominantly acute toxicity of the peptide.

### Vpr induces apoptosis in glioma cells

U251 and U87 cells were exposed to 5 μM Vpr for 36 h and activity of the effector caspases 3 and 7 was measured as an indicator of apoptosis. In parallel, the amount of viable cells was determined at each time point using an ATP-dependent luminescence assay. In U251 cells, caspase activity was increased after 12 h and reached a maximum of 3-fold increase after 24 h (t-test 0 h vs. 24 h: p < 0.05) indicating early apoptosis (Figure 1c). The fraction of vital U251 cells decreased analogously, thus the decrease in caspase activity noted after 24 h was likely caused by a general loss of cells (Figure 1d). Vpr did not cause an increase in activity of caspase 3/7 in U87 cells indicating absence of apoptosis. However, cell counts decreased within 24 h compared to untreated controls, indicating a predominantly cytostatic than pro-apoptotic activity of Vpr in these cells.

### Vpr and TMZ show weak additive effects

**Figure 2:**
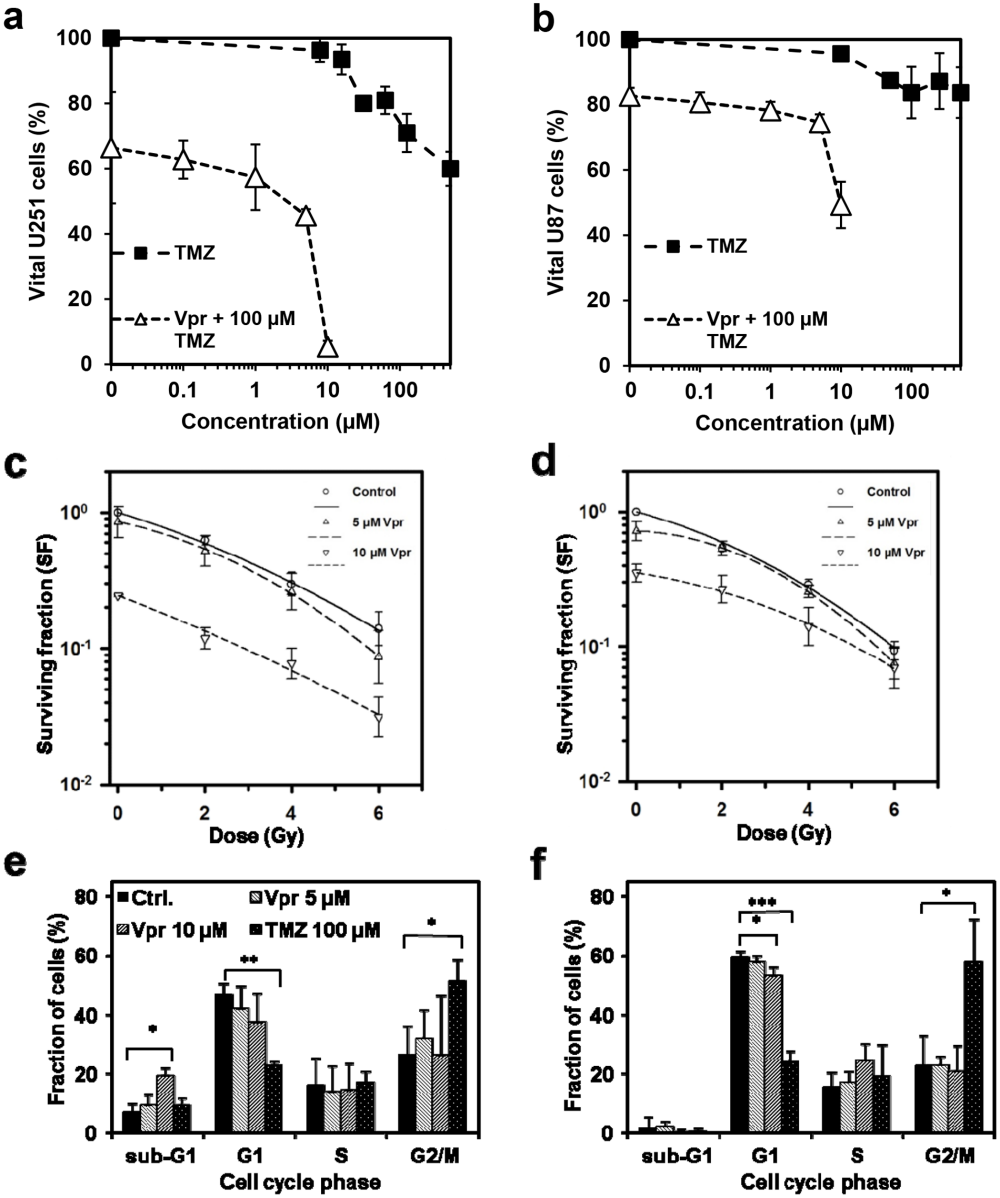
Vpr shows additive effects in combination with TMZ and RT and inhibits clonogenic survival in glioma cells Cell viability after treatment of U251 **a.** and U87 cells **b.** for 72 h with Vpr alone (0.1 – 10 μM), TMZ alone (10 – 500 μM) or with a combination of Vpr and TMZ (100 μM). The DMSO concentration in all samples was adjusted to the highest concentration used in TMZ samples (0.5 %). **c.** Colony formation assays demonstrate clonogenic survival of U251 and U87 cells **d.** after combined treatment with Vpr and radiotherapy. Results are presented as geometric means (n = 3) ± SD. **e, f.** Cell cycle distributions after 48 h in U251 and U87 cells. TMZ was used as a positive control for induction of G2 cell cycle arrest. Results from three independent experiments are presented as means ± SD or representative figures, respectively. *p<0.05; **p<0.01; ***p<0.001

We next assessed the efficiency of a combined modality treatment with Vpr and TMZ (Figure 2a and 2b). Here a concentration of 100 μM of TMZ, which is roughly twice the level achieved *in vivo* [29], resulted in only moderate cell kill within 72 h in U251 (portion of vital cells at 100 μM TMZ: 71±6 %) and U87 cells (84±8 %). Combined application of TMZ (at a concentration of 100 μM) and Vpr resulted in only mildly elevated cell kill and revealed a weak additive rather than a synergistic effect of Vpr and TMZ in U251 (CI_m_ = 0.8±0.4, mean ± SEM) and U87 cells (CI_m_ = 0.8±0.2, mean ± SEM).

### Vpr inhibits clonogenic survival and acts additively with irradiation

In combined treatment with RT (2-6 Gy), Vpr alone (0 Gy) reduced clonogenicity both in U251 and U87 cells (SF for U251 (geometric means ± SEM): 0.85±0.26 at 5 μM, 0.24±0.004 at 10 μM; SF for U87: 0.71±0.11 at 5 μM; 0.35±0.05 at 10 μM; Figure 2c and 2d). Combination therapy with irradiation and Vpr also revealed additive effects (U251: CI_m_ = 1.19±0.25; U87: CI_m_ = 1.14±0.24).

### Vpr does not inhibit cell cycle progression

To clarify whether Vpr may also block the G2/M phase in glioma cells, we performed Nicoletti cell cycle assays using Vpr concentrations of 5 and 10 μM (Figure 2e and 2f; Supplementary Table 1). TMZ, which is an efficient G2/M blocker, served as control (100 μM; [30]). Cells treated with TMZ showed a significant reduction in G1 (U251: p=0.004; U87: p<0.0001) and an increase in G2/M portions (U251: p=0.011; U87: p=0.028). Vpr did not cause a G2/M arrest in the tested cell lines, however, the G1 fraction decreases slightly but not significantly in U251 cells and significantly in U87 cells (60±2 vs. 53±3 %, p=0.027) after treatment with 10 μM Vpr. Simultaneously, the sub-G1 fraction (apoptotic cells) of U251 cells increased after Vpr treatment (10 μM Vpr: 6.9±1.1 vs. 19.2±6.8 %, p=0.037), whereas the sub-G1 fraction of U87 cells remained unchanged.

### Vpr acts independently of MGMT expression

**Figure 3:**
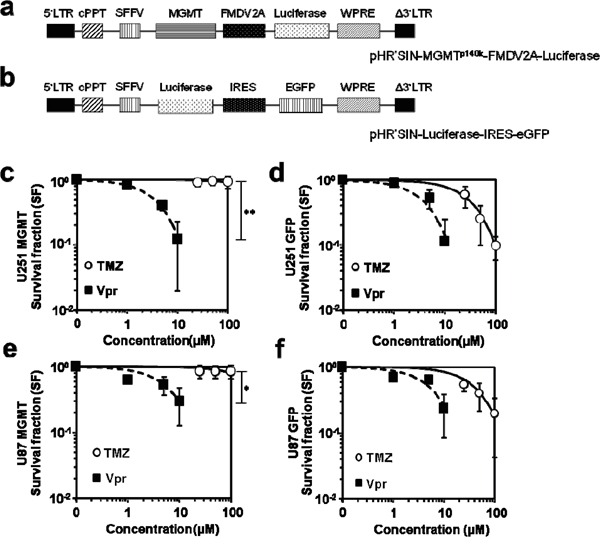
Vpr effects are not recovered by MGMT **a.** Schematic representation of the lentiviral vector used for expression of MGMT, **b.** shows the corresponding vector for eGFP expression (control). **c.** Clonogenic survival of U251-MGMT and **e.** U87-MGMT cells after exposure to TMZ or Vpr. **d.** Survival of (wild-type) U251 and U87 **f.** cells after exposure to TMZ or Vpr. Legend: LTR, long terminal repeat; Δ3‘LTR, 3‘LTR with deleted U3 region; cPPT, central polypurine tract; SFFV, spleen focus-forming virus LTR sequences; WPRE, woodchuck hepatitis virus post-transcriptional regulatory element; MGMT, O^6^-Methylguanine-DNA-Methyltransferase; EGFP, enhanced green fluorescent protein; FMDV2A, 2A element of foot and mouth disease virus; IRES, internal ribosomal entry site; *p<0.05; **p<0.01.

We used lentiviral vectors to induce MGMT or GFP (control) over-expression to evaluate whether MGMT may have a role in Vpr-induced damage repair (Figure 3a and 3b). To also account for late cytotoxic effects of TMZ [31], we used the colony formation assay. As expected, MGMT overexpression resulted in marked resistance of U251 cells towards TMZ (SF of U251-MGMT (geometric means ± SEM): 0.93±0.11 at 100 μM TMZ vs. 0.10±0.02 for U251-GFP; p<0.01; Figure 3c). Similar data were obtained for U87 cells (SF of U87-MGMT (Geometric means ± SEM): 0.85±0.19 vs. 0.19±0.07 for U87-GFP; p<0.05; Figure 3d). In turn, following treatment with Vpr, both MGMT and GFP-transduced cell lines showed decreased survival without statistically significant differences in the fractions, demonstrating that Vpr effects are entirely independent of MGMT expression levels (Figure 3e and 3f).

### Intravenous application of Vpr is non-toxic to mice

A main prerequisite for future application of the peptide is tolerability *in vivo*. This was tested in a classical 3+3 dose escalation regimen in close analogy to clinical phase I trials (Supplementary Figure 2a). Considering that i) therapeutic concentrations range between 5-10 μM, ii) a NOG mouse has approximately 1-1.5 ml total plasma volume and iii) Vpr consists of mostly hydrophilic amino acids, we initially tested a daily dose of 3×5 mg/kg Vpr per mouse. Following one case of death (which could not be clarified by necropsy) in the 3×5 mg/kg group, a further cohort of 3 mice had to be treated with the same dose (Supplementary Figure 2b). As no more events or deaths occurred, the dose was escalated to 3×10 mg/kg per day and finally to 3×20 mg/kg per day. At the highest dose level (3×20 mg/kg), all mice developed skin irritations in the form of an asymptomatic livid tail discoloration distal to the injection site within 7-10 days after Vpr injection (Supplementary Figure 2c). Blood workups after 2 weeks included renal and liver function tests as well as electrolyte levels and revealed no differences to reference laboratory values of untreated animals (Supplementary Figure 2d). Due to the skin irritations, Vpr was given via an osmotic pump system at 60 mg/kg over one week in all further experiments.

### Intravenous Vpr induces central tumor necrosis, delays tumor growth and prolongs survival *in vivo*

To test whether Vpr has anti-glioma activity *in vivo*, we employed an orthotopic xenograft model of human high-grade glioma. Following engraftment of U87 cells in the basal ganglia of NOG mice, all animals were continuously monitored by micro-CT, once a tumor was detected (defined as day 0). The animals either underwent observation only, intravenous Vpr treatment over 1 week using an implanted osmotic pump or irradiation with 3×5 Gy every other day within one week after day 0. The delivery of Vpr via an osmotic pump was chosen to circumvent tail vein injection after the three cases of local tissue reaction (see above). The median tumor volumes at day 0 ranged between 0.26, minimum 0.05, maximum 0.80 mm^3^ (3×5 Gy group), 0.44, minimum 0.04, maximum 1.30 mm^3^ (untreated group), and 0.34, minimum 0.31, maximum 2.34 mm^3^ (Vpr group) and were not statistically different among the three groups (Supplementary Figure 3a; all p > 0.18; Mann-Whitney-U-test). Furthermore, tumor volume on day 0 did not correlate with survival time (Supplementary Figure 3b), indicating comparable initial conditions for all three groups.

**Figure 4:**
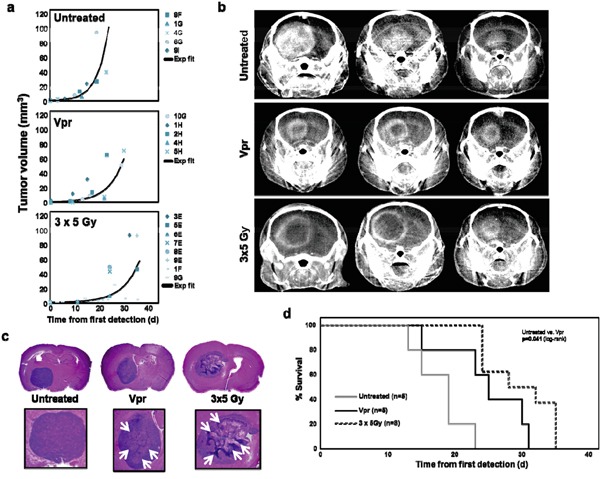
Vpr is effective in a murine orthotopic glioma xenograft model **a.** Shown are the individual tumor volumes (in mm^3^) as a function of time for untreated mice (= control group), mice treated with Vpr or with irradiation (3×5 Gy). An exponential growth curve model was used to fit the data for each mouse with correlation coefficients (adjusted R^2^) in the range from 0.719 to 1.000. The lines represent the exponential fit curves for each data set. Correlation coefficients (adjusted R^2^) of these curves were 0.812 (untreated animals), 0.763 (Vpr) and 0.793 (3×5 Gy). **b.** Exemplary coronal images of contrast-enhanced micro-CT scans of three mice from each group. **c.** Histological workup of murine brains in untreated, Vpr-treated and irradiated mice. The upper panel shows 7 μm-slices of HE-stained murine brains, the lower panel shows a zoom on the corresponding tumor. White Arrows (→) indicate necrotic regions. Of note, the three samples were obtained at different time points, i.e. when animals were exhibiting symptoms and were sacrificed. **d.** Survival curves of the three groups. The log-rank test (see Supplemental Table 2 for details) was used for comparisons of survival curves.

When comparing tumor growth curves, we observed that constant infusion of Vpr over 1 week resulted in a delay of intracranial tumor growth as compared to untreated animals (Figure 4a). While the control group showed homogenous contrast enhancement of the complete tumor in regular CT scans, all animals treated with Vpr or irradiation (3×5 Gy) presented with a central irregular non-enhancement (Figure 4b), suggesting therapeutic effects on the tumors. Consistent with these findings, histological analyses of murine brains showed tumors with central tumor necrosis in Vpr-treated and irradiated mice which was not observed in this extent in untreated mice (Figure 4c). Finally and most importantly, both Vpr and irradiation significantly prolonged overall survival of tumor-bearing mice (p_Vpr_=0.041, and p*_3×5 Gy_* <0.001; log-rank test; Supplementary Table 2, Figure 4d). Of note, compared to all other mice in the trial, the Vpr-treated mouse that died at day 15 showed the largest initial tumor volume (2.3 mm^3^ at day 0; Supplementary Figure 3b).

### Combined treatment with irradiation and Vpr is not superior to Vpr monotherapy

To assess whether Vpr is synergistic to radiotherapy, we treated tumor-bearing mice with Vpr (60 mg/kg over one week) and fractionated irradiation (5×3 Gy). Although this treatment arm contained the fewest number of mice (n=4) and resulted in the longest survival of one animal observed within the whole experiment (41 days after first detection), we did not detect an statistically significant difference in overall survival compared to Vpr monotherapy (Supplementary Figure 4).

## DISCUSSION

The current study shows that the HIV-derived protein Vpr induces apoptosis in glioma cells, acts independently from MGMT expression status, is tolerated well when given intravenously, delays intracranial tumor growth and prolongs survival in a relevant pre-clinical model of high-grade glioma. Therefore, it has all the traits of an potentially useful agent for the treatment of GBM.

In the light of a rather frustrating decade passing without any significant new therapy option for GBM (and even less for unmethylated GBM), there is an urgent need for more effective therapies to improve poor outcomes in this lethal disease. Novel compounds should i) be able to cross the blood-brain barrier (BBB), ii) be active against glioma cells *per se* and possibly also in those resistant to TMZ and iii) be tolerated adequately at therapeutic doses. A minor prerequisite not specifically related to GBM is a non-complex synthesis and thus an affordable market price. In our opinion Vpr, a peptide encoded by the human immunodeficiency virus (HIV), potentially fulfils all of the mentioned requirements.

In GBM, drugs may not necessarily need to actively “cross” the BBB as these tumors usually present with a disrupted BBB [32, 33], which is even further aggravated by radiotherapy [34–36]. However, although GBM is characterized by a high local relapse rate, it resembles a (central nervous) systemic disease as multiple tumor cells are occultly disseminated throughout the brain at the time of diagnosis [37]. Therefore, suitable antiglioma drugs must be also capable of targeting dispersed tumor cells located in areas with an intact BBB. Vpr may fulfil these criteria as it efficiently crosses cellular membranes and is rapidly internalized [38–40]. Vpr is detectable in significant amounts in cerebrospinal fluid (CSF) of HIV-positive patients, indicating that either infected cells (lymphocytes or microglia) or viral particles cross the BBB, or blood–CSF barrier transition of Vpr in the choroid plexus takes place [20, 41].

The second prerequisite is also fulfilled by Vpr: several reports have demonstrated that Vpr has proven to be toxic for a variety of tumor cell lines *in vitro*, including neuroblastoma and grade III astrocytoma cell lines [8, 20–22]. For the first time, we here showed that the peptide was not only active *in vitro*, but also in a murine orthotopic *in vivo* model. The protein delayed tumor growth as indicated by micro-CT analyses and prolonged survival. It is noteworthy that we measured total tumor volumes, which included the contrast-enhancing parts, as well as the non-enhancing parts (necrotic areas) of the tumors. If we had decided to consider only contrast-enhancing parts (as suggested by RANO criteria), the differences in measured tumor sizes between animals treated with Vpr or irradiation and the untreated control animals would have been even more prominent.

Although the exact mode of action of Vpr is not clarified yet, it has been described that Vpr induces double strand breaks [15, 16] causing a cisplatin- and alkylator-like cellular damage signature [15, 18]. Here, we also showed that Vpr inhibited clonogenic survival, indicating a considerable genomic damage upon drug exposure. Alkylators such as TMZ cause DNA damage and clonogenic cell death by transferring methyl groups to DNA, which can be efficiently and timely removed by MGMT. As the number of viable cells in our MTT assays did not decrease with prolonged exposure to Vpr (the maximum observation period was 72 h), we assume that Vpr rapidly penetrates the cells and subsequently induces irreversible genomic damage. As we did not see any effect of MGMT overexpression on survival of U87 and U251 cells, genomic instability caused by Vpr is likely entirely distinct from this mechanism.

We did not observe Vpr-induced G2/M arrest in U87 and U215 and, consistent with this, clonogenic assays revealed no radiosensitizing effects. One reason may be that we used a truncated version of Vpr lacking the n-terminus. Although there is consensus that apoptosis is mediated by the c-terminal end [42, 43], the region of Vpr causing cell cycle arrest is to date controversially discussed [44, 45]. A further reason may be that Vpr induced cell cycle effects are not unspecific and dependent on different cellular backgrounds. Most of the studies that demonstrated efficient G2/M arrest were performed to explore HIV pathogenicity and, consequently, the cell lines used to evaluate this effect were derived from lymphomas or leukemias – tumors entirely different to glioblastoma [11, 12].

An indispensable characteristic of a useful drug is adequate tolerability of therapeutic doses, which, due to the apparent high and unspecific toxicity of Vpr, was a major concern. Considering that acutely infected patients with actively replicating HIV have peak plasma levels of Vpr, and patients with chronic replication show 24-hour exposure to the peptide [46], we assumed that intravenous application of the peptide is tolerated. In our dose escalation study, local tissue irritation occurred at highest dose levels (3×20 mg/kg/d) and one case of death was noted at the lowest dose (3×5 mg/kg/d). Local tissue irritation or even necrosis is also commonly observed in the clinical setting after unintended paravenous injection of chemotherapeutics. It is unlikely that all three mice were incorrectly injected in our study and thus we decided to apply the peptide via an osmotic pump with a three-fold reduced dose (60 mg/kg over one week) in the therapeutic setting. Although the cause of death could not be clarified by necropsy, the immunodeficient NOG strain is known to be a rather susceptible strain and unclear deaths also occur frequently in untreated NOG mice.

A possibly relevant concern which was not assessable in our model, is the yet undefined link between Vpr and AIDS-related dementia. Vpr has been shown to exert indirect cytotoxic effects by inducing expression of neurotoxic cytokines in astrocytic and microglial cells [20]. Although this could potentially improve antitumoral responses, it may cause or worsen neurodegeneration with the risk of neurocognitive impairment [47]. However, the overall survival of GBM patients is poor and this specific side effect would be only of significance in a small portion of long-term survivors.

A further advantage of Vpr therapy would be a relatively low market price. Chemically, Vpr as used in this study (the C-terminal fragment Vpr_52-96_) resembles a simple peptide, roughly of the size of insulin (51 aa) and less than a third of the size of erythropoietin alpha. It is soluble in water and stable at 4°C for at least one month (Jens Kuebler, unpublished results). Thus, due to the enormous progress in recombinant DNA technology, large-scale production of recombinant peptides has become feasible at reasonable prices.

Taken together, this study provides evidence that Vpr causes both acute and long-term cytotoxic effects in glioma cells *in vitro* and *in vivo*. As the protein acts independently from MGMT expression status of the tumor cells, it might become a useful substance in the treatment of glioblastoma multiforme.

## MATERIALS AND METHODS

### Viral protein R and alkylating agents

The c-terminal portion of the Viral protein R (Vpr_52-96_, [N-]GDTWAGVEAIIRILQQLLFIHFRIG CRH SRIGVTRQRRARNGASRS[−C]), which contains the active (pathogenic) domain of the viral peptide [40, 48, 49], was used in the reported experiments. The peptide was commercially synthesized (Caslo ApS, Lyngby, Denmark), enriched to >99 % purity and delivered as lyophilized trifluoroacetate salt (powder). The lyophilized peptide was dissolved in sterile water and stock solutions were stored at 4°C. TMZ and O^6^-BG were dissolved in DMSO. BCNU (bis-chloroethylnitrosourea/Carmustin) was dissolved in 100% ethanol. Stock solutions were stored at −20°C. All chemotherapeutic drugs were purchased from Sigma-Aldrich (Munich, Germany).

### Cells and cell culture

All cell lines described here were obtained from ATCC (Manassas, VA) and thus no authentication was done. The human glioblastoma/astrocytoma cell line U251 was cultured in RPMI-1640 medium (Biochrom AG, Berlin, Germany). The human high-grade glioma cell line U87, the human embryonic kidney cell line 293T and the human fibrosarcoma cell line HT1080 were cultured in DMEM (Dulbecco's modified Eagle's medium; Biochrom). All media were supplemented with 10% FCS, penicillin (100 IU/ml) and streptomycin (100 μg/ml). Cells were cultured at 37°C and 5% CO_2_.

### Construction of lentiviral vectors for MGMT, luciferase and GFP expression

For the construction of *pHR'sIN-MGMT^p140k^-FMDV2A-Luciferase,* the sequence of the luciferase reporter gene was amplified from the plasmid pGL3-basic (Promega, Madison, WI, USA). Overhang PCR was used to create an additional *Apa* I restriction site upstream of the luciferase sequence. The following primers (Metabion, Steinkirchen, Germany) were used: Luciferase forward 5′-GCAAGCTTGGGCCCATGGAAGACGCCAAAAAC ATAAAG-3′; Luciferase reverse 5′-CGTGTACATCGACTGAAATCCCTGGTAATCCG-3′. The amplified luciferase fragment was then inserted into MGMT^p140k^-FMDV2A [50] via *Apa*I and *Xba* I. The amplified products were checked by Sanger sequencing (GATC Biotech AG, Konstanz, Germany). Next, the fragment *MGMT^p140k^-FMDV2A-Luciferase* was inserted into the lentiviral plasmid *pHR'sIN-cPPT-SEW* [51] using *Bam*HI and *Xba* I replacing the coding sequence of eGFP (a second *Xba* I site behind the U3LTR was deleted in advance), resulting in *pHR'sIN-MGMT^p140k^-FMDV2A-Luciferase.* For the transduced controls, we used the vector *pHR'SIN-Luciferase-IRES-eGFP*, which is based on *pHR'SIN-SNAI2* [52] and in which the cDNA of *SNAI2* upstream of the IRES-EMCV element was replaced by the cDNA of luciferase after digestion with *Bam*HI and *Xba* I.

### Production of lentiviral supernatant and determination of viral titers

Lentiviral supernatant was produced as described before [51, 53]. In brief, 293T cells were transfected with the lentiviral plasmids *pHR'sIN-MGMT^p140k^-FMDV2A-Luciferase* or *pHR'SIN-Luciferase-IRES-eGFP* and the two packaging plasmids *pCMVΔR8.91* and *pMD.G* in the presence of Metafectene (Biontex, Martinsried/Planegg, Germany). Lentiviral supernatants were then collected after 48 h, filtrated (0.45 μm pore-size filter; Millipore, Carrigtwohill, Ireland) and concentrated using Vivaspin filters (100.000 MWCO; Sartorius, Goettingen, Germany). Aliquots of the viral supernatants were shock-frozen and stored at – 80°C. For the determination of viral titers, HT1080 cells were transduced with serial dilutions of the viral supernatants in the presence of polybrene (8 μg/ml; Sigma-Aldrich). Viral titers for the vector *pHR'sIN-Luciferase-IRES-eGFP* were determined by fluorescence-activated cell sorting analysis for eGFP marker gene expression. Titers of the *pHR'sIN-MGMT^p140k^-FMDV2A-Luciferase* vector were determined via a Luciferase Assay (Promega, Fitchburg, Wisconsin, USA) according to the manufacturer's protocol, whereas the intensities of luminescence were compared to HT1080 cells which were transduced in serial dilutions with the control vector *pHR'sIN-Luciferase-IRES-eGFP* at a multiplicity of infection (MOI) of 10.

### Transduction of cells

To generate MGMT-expressing U251 and U87 cells, both cell lines were transduced in presence of polybrene (8 μg/ml) with the MGMT vector *pHR'sIN-MGMT^p140k^-FMDV2A-Luciferase* at a MOI of 1 and the control vector *pHR'sIN-Luciferase-IRES-eGFP* at a MOI of 10. The GFP-positive population of the control cells U251-GFP and U87-GFP were separated via flow cytometry (BD FACSAria I, Becton Dickinson, Heidelberg), and the sorting process was repeated after 7 days. Cells transduced with the MGMT-containing vector were subjected to selection with O^6^-BG (100 μM) and BCNU (150 μM). To validate successful selection of MGMT-expressing cells, the luciferase expression levels were determined using the Luciferase Assay System (Promega).

### Irradiation of cells

Cells were irradiated with various doses of 6 MV X-rays from a conventional linear accelerator (Versa HD^TM^, Elekta AB, Stockholm, Sweden). Irradiation was performed in T25 flasks (colony formation assay) with a field setup published previously [54]. In combined modality treatments (together with Vpr or TMZ) or control treatments (DMSO), irradiation was performed 6 hours after the addition of the corresponding substance.

### MTT assay

Cells were seeded in 96-well plates (1 to 5×10^3^/well) in 80 μl medium with supplements. Then drugs were added in a volume of 20 μl to the following final concentrations: Vpr*:* 0.1 μM to 20 μM; TMZ: 10 μM to 500 μM. DMSO concentration in all samples was adjusted to the highest concentration given by TMZ samples (<0.5 %). 20 μl MTT (3-(4,5-dimethylthiazol-2-yl)-2,5-diphenyltetrazolium bromide; 5 mg/ml) was added 24, 48 or 72 h after addition of drugs and the plate was incubated at 37°C for 4 hours. Formazan crystals forming after addition of MTT were dissolved with 100 μl SDS (sodium dodecyl sulfate; 10% in 10 mM HCl) overnight. Thereafter, absorbance was measured at 590 nm (690 nm reference) using a microplate reader (Tecan Infinite M200, Tecan AG, Männedorf, Switzerland). Results were normalized to controls and shown as percent values.

### Colony forming assay

Cells were seeded in T25 Flasks (Falcon, Becton-Dickinson) in the desired densities (2×10^2^ to 6×10^3^) into 5 ml medium with supplements and in triplicates. Drugs (Vpr, TMZ and/or DMSO) were added to final concentrations (Vpr 0 μM to 10 μM; TMZ 0 μM to 100 μM). The concentration of DMSO was adjusted to the highest concentration given by TMZ samples (0.1%). Cells were incubated for 6 h at 37°C and irradiated with 6 MV X-rays from a linear accelerator (Synergy, Elekta, Stockholm, Sweden) at doses up to 6 Gy. Directly after the irradiation, the medium was removed and cells were washed carefully with PBS. Thereafter, fresh medium with supplements was added and cells were allowed to grow colonies for 14 to 21 days. In experiments without irradiation (MGMT/eGFP cells lines), the medium was exchanged after 6 h of treatment and cells were allowed to grow colonies. Colonies (defined as cluster of >50 cells) were fixed, stained and counted as described previously [55].

### Apoptosis and cell viability assays

To detect apoptosis, the Caspase-Glo® 3/7 assay (Promega, Fitchburg, Wisconsin, USA) was used according to the manufacturer's instructions. In brief, 5×10^3^ cells/well were plated in 96 well plates. Vpr (5 μM) was then added and plates were incubated at 37°C until measurements were performed at 0, 6, 12, 18, 24, 30 and 36 h after Vpr addition using a microplate reader (Tecan infinite M200). To quantify (remaining) viable cells in the apoptosis assays, we used the Cell Titer-Glo® Luminescent Cell Viability Assay (Promega) with identical numbers of plated cells and identical treatments as performed in the caspase assay.

### Cell cycle analysis

To determine distribution of cell cycle phases and the fraction of apoptotic cells (sub-G_1_) after treatment with Vpr we used flow cytometry as described previously [56]. Specifically, 1-2×10^6^ U251 or U87 cells were plated in T-75 cm^2^ flasks and cells were allowed to adhere. Next, Vpr (5 μM or 10 μM) or TMZ (100 μM, DMSO 0.1%) was added. Cells were incubated for 6 h at 37°C and medium was then exchanged. Cells were harvested after 48 h, fixed overnight in 70 % ice-cold ethanol, washed again and resuspended in PBS. Then, RNase A (2 mg/ml, Sigma-Aldrich) and propidium iodide (1 mg/ml, Sigma-Aldrich) was added and, after incubation for 30 min, cells were analyzed via flow cytometry (BD FACS Canto II). The measurement was analyzed using FlowJo 7.6.5 software (Tree StarInc, Ashland, OR, USA).

### Orthotopic murine glioma model

Immunodeficient 6 to 8 weeks old female NSG mice (NOD.Cg-*Prkdc^SCID^*Il2rg*^tm1Wjl^*/SzJ, The Jackson Laboratory, Bar Harbor, Maine, USA) were used for orthotopic tumor implantation. Prior to surgery, all mice were anesthetized by subcutaneous injection of MMF (0.5 mg/kg medetomidine, 5 mg/kg midazolam and 0.05 mg/kg fentanyl) and positioned in a stereotactic frame (TSE Systems, Bad Homburg, Germany) under an operating microscope. After drilling of a 0.5 mm burr hole 1 mm anterior to the bregma and 3 mm lateral to the midline, 2×10^6^ U87 cells were injected with a glass syringe (Neuros Syringe, Hamilton) using a 33G blunt needle into the basal ganglia over approximately 10 min [57, 58]. For postoperative pain management, all animals received 200 mg/kg metamizole p.o. (added to tap water). All mice were sacrificed if they showed neurological symptoms or more than 20 % of body weight loss. All animal experiments were approved by the local and governmental authorities.

### Treatment with Vpr

For tolerance testing, we used a 3+3 study in analogy to clinical dose finding trials. Due to the mostly acute effects of the peptide observed *in vitro*, we considered an observation period of 14 days to be sufficient to rule out relevant acute toxic effects *in vivo*. Three mice of the first cohort received i.v. injection (lateral tail vein) with 5 mg/kg of water-dissolved Vpr three times on days 0, 2 and 4 and were closely followed for the development of signs of toxicity up to day 14. Thereafter, the mice were sacrificed and blood was subjected to liver (LFT) and renal function tests (RFT). If no toxicity was noted, the dose was doubled to 3×10 mg/kg/d and then to 3×20 mg/kg/d. If a mouse showed pathological LFT, RFT or died, a further group of 3 mice received the same dose. If no signs of toxicity were observed, the dose was escalated to the next dose level.

For tumor treatment with Vpr, tumor-bearing mice were anaesthetized using MMF (see above) and an osmotic minipump (ALZET 2001, DURECT Corp, Cupertino, CA) was placed subcutaneously with a catheter (MJC-AL, DURECT) implanted into the right jugular vein. The pumps were filled with 170 μl of water-dissolved Vpr to deliver a dose of 60 mg/kg over a period of 1 week (1 μl/h). Before implantation and after explanation, all pumps were weighed to ensure correct treatment. Mice bearing pumps with evident catheter clotting or irregular weight were excluded from further analyses.

### Irradiation of mice and combined radiotherapy and Vpr treatment

Irradiation was performed under anesthesia (MMF, see above) with three coplanar beams in a fractionated regimen of 15 Gy delivered in three fractions of 5 Gy by a small-animal irradiation device capable of image-guided radiotherapy [59]. For validation of adequate target volume coverage we used Gafchromic® films mounted between RW3 plates (Supplementary Figure 1). In mice treated with combined therapy (Vpr and radiotherapy), irradiation was performed one day after implantation of the osmotic pump.

### *In vivo* monitoring of tumor growth

Starting two weeks after engraftment, animals were screened weekly for intracranial tumors using contrast-enhanced micro-CT (Kirschner *et al*., *in re-submission*; [59]. Briefly, 300 μl of a iodine-based contrast agent (Iomeprol; Imeron® 300, Bracco Imaging Group, Germany) were injected via the lateral tail vein and CT-imaging was performed within 5 min as described before [60, 61]. Tumor volumetry of micro-CT images was performed by contouring total tumor volumes (contrast-enhancing + non-enhancing regions) using OsiriX Freeware [62].

### Statistics and survival analysis

Results of all *in vitro* assays are presented as means of at least three independent experiments together with the corresponding standard deviations. For detection of statistical differences in all *in vitro* assays, an unpaired two-sided t-test was used. To account for multiple testing in cell culture experiments, we adjusted p values using Bonferroni correction. For calculation of IC50 values and for evaluation of combination effects, we used the method of Chou and Talalay [63] available in the CalcuSyn software suite (Biosoft, Cambridge, UK). Mean combination index (CI_m_) values were calculated and assessed whereby CI values greater than 1, equal to 1 or smaller than 1 indicated antagonism, additivity or synergism. Surviving fractions (SF) for irradiation experiments were fitted by the linear-quadratic model (ln(SF) = −(αD + βD^2^) by nonlinear regression using SigmaPlot v11 (Systat Software GmbH, Erkrath, Germany) [55].

Tumor volumes at day 0 in the different treatment groups were compared using a Mann-Whitney-U test. For comparisons of *in vivo* tumor growth, we used an exponential growth curve model to fit the CT data for each mouse. No formal statistical testing was performed, owing to the relatively small number of animals and the frequency of missing CT data at different time points.

Overall survival (OS) of mice was measured from the date of diagnosis (defined as the day on which a tumor was detected in a CT scan) until the date of death and the log-rank test (Mantel-Cox) was used for comparisons of survival. All statistical analyses of *in vivo* data were performed using SPSS version 22 (IBM Corp., Armonk, NY, USA).

## SUPPLEMENTARY FIGURES AND TABLES


