# Supplementary material for: The HIV-derived protein Vpr52-96 has anti-glioma activity in vitro and in vivo
Source: Oncotarget. 2016 Jun 2;7(29):45500–12. doi: 10.18632/oncotarget.9787 (PMC5216737; doi:10.18632/oncotarget.9787)
Supplement: Supplementary file 1 [file oncotarget-07-45500-s001.pdf]

# The HIV-derived protein $Vpr_{52-96}$ has anti-glioma activity *in vitro* and *in vivo*

## SUPPLEMENTARY FIGURES AND TABLES

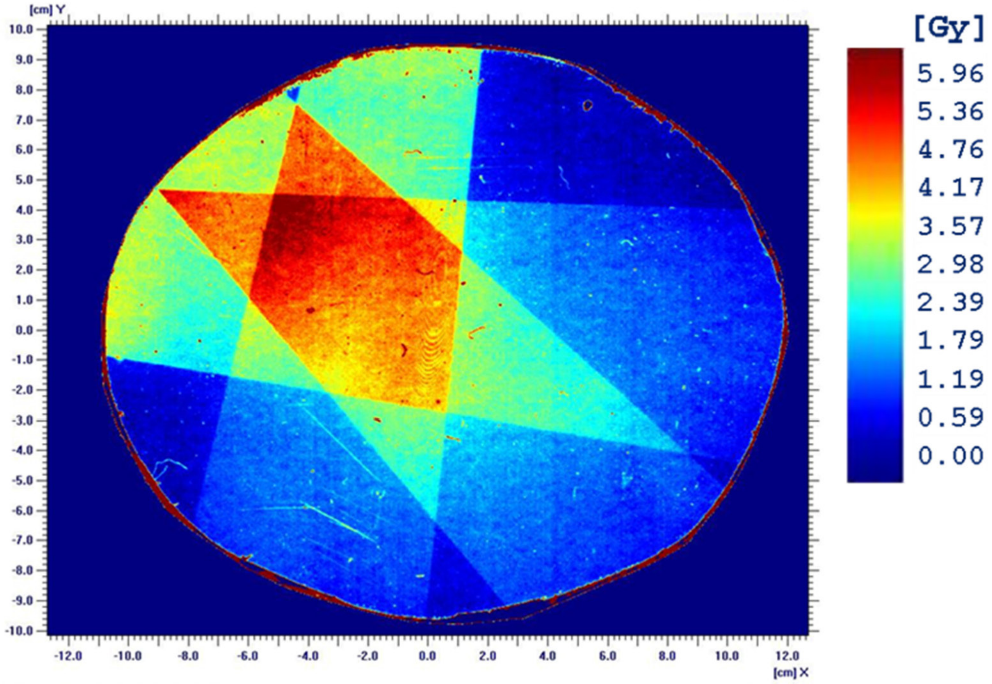


**Supplementary Figure S1: Film validation of the dose distribution.** To validate adequate target volume coverage, GafChromic® films were placed between water-equivalent RW3 slabs and irradiation from three angles was performed up to a total dose of 5 Gy.

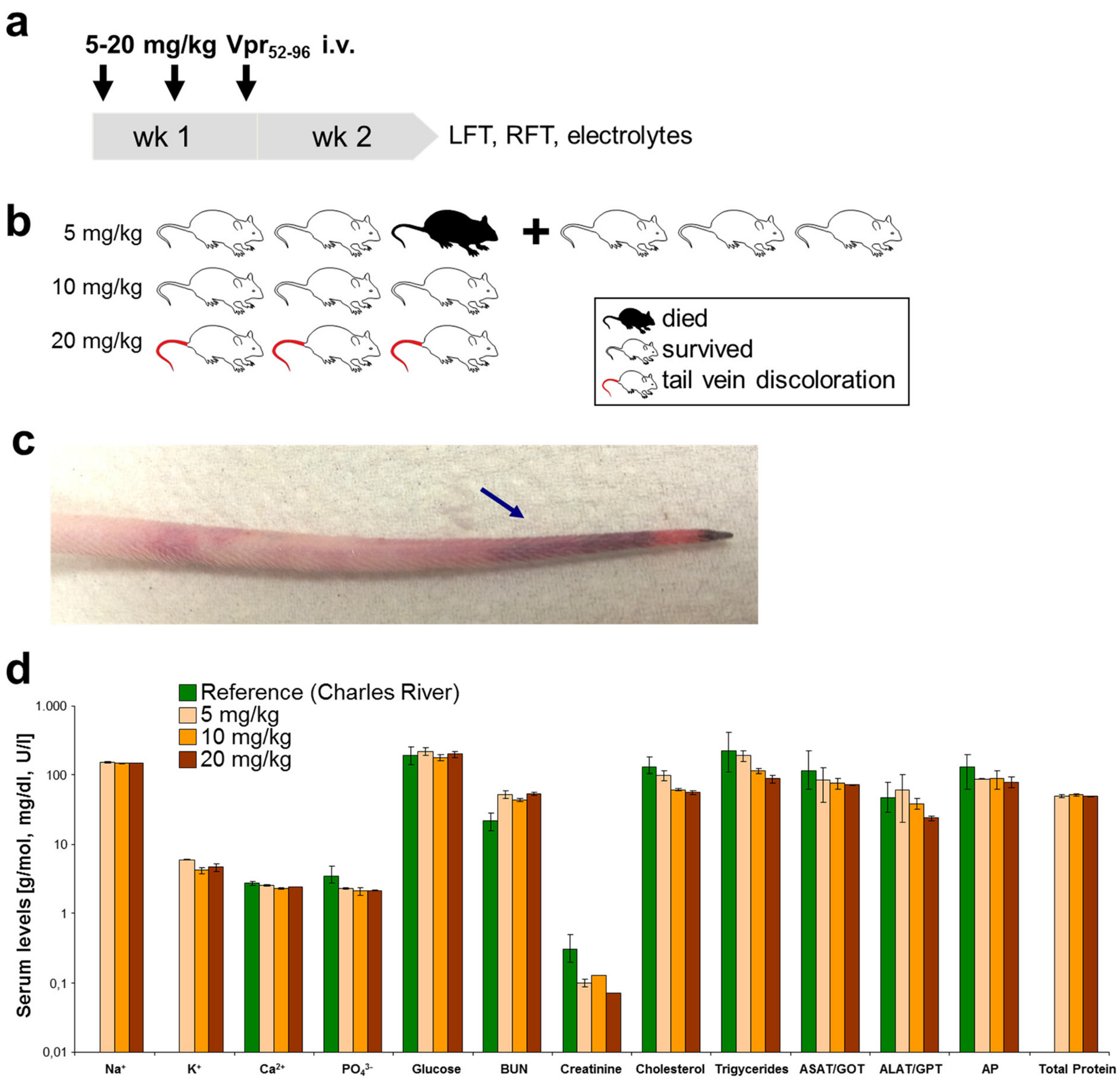


**Supplementary Figure S2: Dose escalation study.** **a.** Schematic outline on thetreatment and analysis regimen. **b.** result of a dose escalation study using a classical 3+3 design. **c.** Exemplary image of a distal tail vein discoloration 6-7 days after Vpr injection. **d.** Blood workups of reference mice (provided by Charles River, Inc.) and of mice treated in the 3+3 dose finding study.

**a**

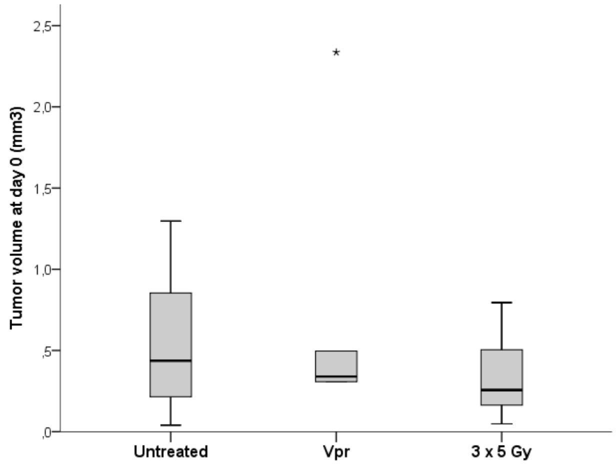


**b**

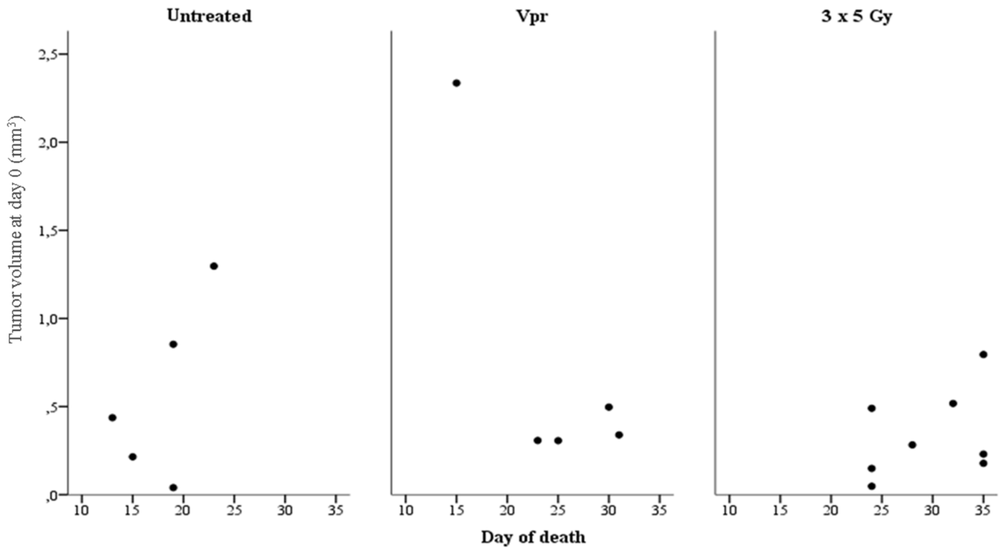


| | Spearman's rho | p |
|---|---|---|
| Untreated | 0.462 | 0.434 |
| Vpr | -0.200 | 0.747 |
| 3 x 5 Gy | 0.401 | 0.325 |

**Supplementary Figure S3: Tumor volumes at day 0. a.** Box-and-whisker plot for tumor volumes at day 0 (day of first detection of tumors). The box indicates the interquartile range, the whiskers indicate the range, and the horizontal line within the box represents the median. Extreme values are plotted as asterisks. **b.** The upper graph shows tumor volumes at day 0 plotted against survival time, the table below shows results of a non-parametric test (Spearman's rho) used to measure the strength of associations.

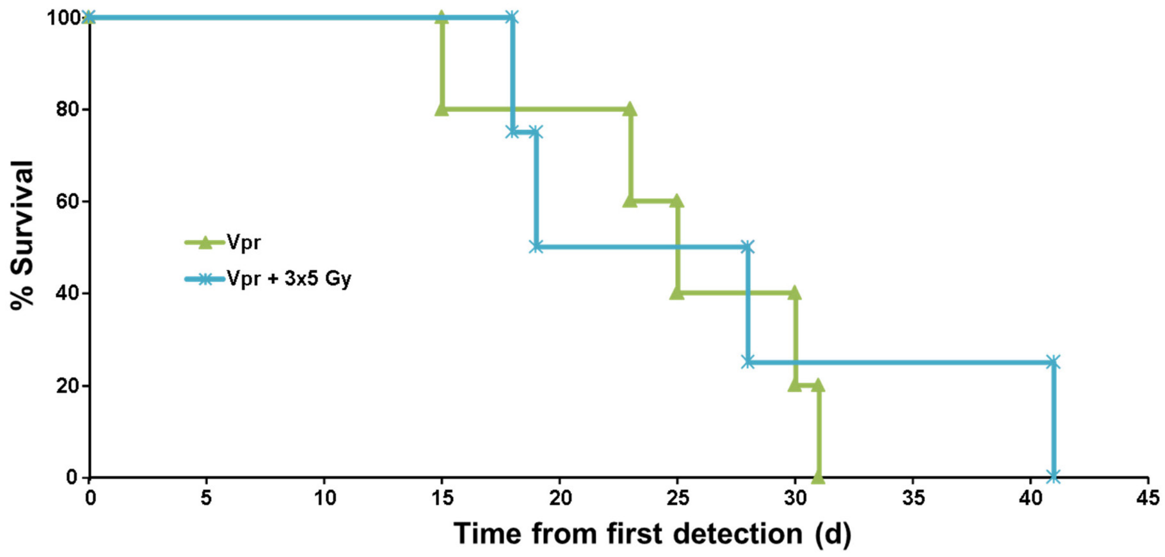


**Supplementary Figure S4: Survival after Vpr monotherapy versus combined treatment with Vpr and irradiation.** Shown are survival curves for Vpr monotherapy (60 mg/kg over 1 week) and combined therapy with Vpr and irradiation (3 x 5 Gy every second day). There was no statistical difference between both survival curves.

**Supplementary Table S1: Cell cycle distributions after Vpr and TMZ treatment**

| | | sub-G1 (%) | | G1 (%) | | | S (%) | G2/M (%) | |
|---|---|---|---|---|---|---|---|---|---|
| U251 | Control | 6.9 ± 1.1 | | 46.5 ± 6.8 | | | 16.1 ± 3.3 | 26.5 ± 6.4 | |
| | Vpr 5 µM | 7.4 ± 4.2 | * | 42.1 ± 6.5 | ** | | 13.9 ± 5.8 | 31.8 ± 5.0 | * |
| | Vpr 10 µM | 19.2 ± 6.8 | | 37.4 ± 2.7 | | | 14.5 ± 6.3 | 26.3 ± 8.4 | |
| | TMZ 100 µM | 9.2 ± 2.4 | | 22.8 ± 1.1 | | | 16.9 ± 3.8 | 51.3 ± 7.2 | |
| U87 | Control | 1.8 ± 3.4 | | 59.5 ± 1.8 | | | 15.5 ± 4.7 | 22.8 ± 9.9 | |
| | Vpr 5 µM | 2.1 ± 1.8 | | 57.6 ± 2.4 | * | *** | 17.0 ± 3.7 | 22.8 ± 3.1 | * |
| | Vpr 10 µM | 0.3 ± 0.8 | | 53.1 ± 2.7 | | | 24.3 ± 5.8 | 20.8 ± 8.6 | |
| | TMZ 100 µM | 0.4 ± 0.9 | | 23.9 ± 3.5 | | | 19.1 ± 10.6 | 57.5 ± 14.7 | |

All Experiments were performed three times in triplicates. Shown are mean values ± standard deviations.

**Supplementary Table S2: Comparison of survival**

| | Untreated | | Vpr | |
|---|---|---|---|---|
| | $\chi^2$ | p | $\chi^2$ | p |
| Untreated | | | | |
| Vpr | 4.165 | 0.041 | | |
| 3 x 5 Gy | 14.370 | 0.000 | 2.935 | 0.087 |

This table shows the result of a log-rank test (Mantel-Cox), which was used to compare the survival distributions of the three groups.
